# Myoblast sensitivity and fibroblast insensitivity to osteogenic conversion by BMP-2 correlates with the expression of *Bmpr-1a*

**DOI:** 10.1186/1471-2474-10-51

**Published:** 2009-05-15

**Authors:** Renjing Liu, Samantha L Ginn, Monkol Lek, Kathryn N North, Ian E Alexander, David G Little, Aaron Schindeler

**Affiliations:** 1 Orthopaedic Research & Biotechnology Unit, The Children's Hospital at Westmead, Sydney, NSW, Australia; 2 Discipline of Paediatrics and Child Health, Faculty of Medicine, University of Sydney, Sydney, NSW, Australia; 3 Gene Therapy Research Unit, Children's Medical Research Institute and The Children's Hospital at Westmead, Sydney, NSW, Australia; 4 The Institute for Neuromuscular Research, The Children's Hospital at Westmead, Sydney, NSW, Australia

## Abstract

**Background:**

Osteoblasts are considered to primarily arise from osseous progenitors within the periosteum or bone marrow. We have speculated that cells from local soft tissues may also take on an osteogenic phenotype. Myoblasts are known to adopt a bone gene program upon treatment with the osteogenic bone morphogenetic proteins (BMP-2,-4,-6,-7,-9), but their osteogenic capacity relative to other progenitor types is unclear. We further hypothesized that the sensitivity of cells to BMP-2 would correlate with BMP receptor expression.

**Methods:**

We directly compared the BMP-2 sensitivity of myoblastic murine cell lines and primary cells with osteoprogenitors from osseous tissues and fibroblasts. Fibroblasts forced to undergo myogenic conversion by transduction with a MyoD-expressing lentiviral vector (LV-MyoD) were also examined. Outcome measures included alkaline phosphatase expression, matrix mineralization, and expression of osteogenic genes *(alkaline phosphatase, osteocalcin *and *bone morphogenetic protein receptor-1A) *as measured by quantitative PCR.

**Results:**

BMP-2 induced a rapid and robust osteogenic response in myoblasts and osteoprogenitors, but not in fibroblasts. Myoblasts and osteoprogenitors grown in osteogenic media rapidly upregulated *Bmpr-1a *expression. Chronic BMP-2 treatment resulted in peak *Bmpr-1a *expression at day 6 before declining, suggestive of a negative feedback mechanism. In contrast, fibroblasts expressed low levels of *Bmpr-1a *that was only weakly up-regulated by BMP-2 treatment. Bioinformatics analysis confirmed the presence of myogenic responsive elements in the proximal promoter region of human and murine *BMPR-1A/Bmpr-1a*. Forced myogenic gene expression in fibroblasts was associated with a significant increase in *Bmpr-1a *expression and a synergistic increase in the osteogenic response to BMP-2.

**Conclusion:**

These data demonstrate the osteogenic sensitivity of muscle progenitors and provide a mechanistic insight into the variable response of different cell lineages to BMP-2.

## Background

The conventional view of bone cell differentiation is that pluripotent or multi-potent stem cells commit to an osteoprogenitor lineage and then undergo a smooth, consistent, and well defined transition to produce differentiated osteoblasts. Osteoprogenitors are considered to be committed to an osteochondral lineage once they express the key transcription factor *Runx2/Cbfa1 *[1]. As osteogenic differentiation proceeds, other markers of bone differentiation such as *osterix*, *alkaline phosphatase (AP)*, *bone sialoprotein*, and *osteocalcin (OCN) *are sequentially expressed.

The processes of osteoprogenitor commitment and osteoblast differentiation are both facilitated by members of the bone morphogenetic protein (BMP) protein family [2]. BMP homodimers and heterodimers bind to BMP receptors (BMPRs) on the cell surface that can transduce the concentrations of extracellular BMPs into specific changes in gene expression [3]. BMPR-IA and BMPR-II are reported to be ubiquitously expressed, whereas BMPR-IB is tissue specific [4-6]. While the expression of BMPs in bone development and repair are well described [7,8], the potential for lineage-specific control of BMP signaling by regulating BMPR expression levels has not been explored in detail.

Committed osteoprogenitors are traditionally considered to arise from progenitors within the periosteum (a thin cellular layer covering bone surfaces) and within the bone marrow. In the bone marrow, the bone marrow stromal cells (BMSCs) encompass a heterogeneous population of highly plastic cells that not only support the haematopoietic progenitors, but also contribute to endosteal bone formation [9]. MSCs are multipotent and can be stimulated to differentiate into cells of the osteoblastic, chondrogenic, myogenic, and adipogenic lineages [10]. By necessity, BMSCs must maintain themselves in a relatively undifferentiated state within the bone compartment. Although BMSCs generally remain in the marrow space, osteoprogenitors likely to have originated from the marrow have also been detected in the circulation [11], and it has been suggested that these circulating cells may also contribute to bone formation and repair [12].

We have speculated that other cell types arising from the soft tissues adjacent to bone may also be able to contribute to bone formation and repair [13]. This would presumably occur via transdifferentiation, a process where non-osseous progenitors could be reprogrammed to an osteogenic lineage [14]. Muscle progenitors are an appealing candidate for osteogenic transdifferentiation, as cultured myoblasts readily adopt a bone cell phenotype upon treatment with the osteogenic BMPs [15,16].

There are multiple indirect observations that suggest stem cells situated within muscle can participate in bone formation and repair. Muscle is found adjacent to most bones and can be exposed to bone signals upon injury. Disease states can also alter bone signaling resulting in ossification within muscle tissue, such as in *myositis ossificans *or in the genetic disease *fibrodysplasia ossificans progressiva *(FOP)[17,18]. Moreover, recent evidence would suggest that inhibition of BMP type 1 receptor signaling may be effective in the treatment of FOP [19]. During recalcitrant bone repair, new bone formation is most commonly seen at the muscle-bone interface. Although this implies the presence of osteoprogenitor cells within muscle, it is unclear whether the cells that contribute to the bone healing process arise from muscle progenitors (satellite cells), are associated with the vasculature (pericytes) [20], exist as a small sub-population of multipotent stem cells [21,22], or migrate from the circulation [11].

Numerous *in vitro *and *in vivo *experiments have induced osteogenesis in skeletal myoblasts [15,16,23-29] however, their sensitivity to osteogenic signals relative to other cell types remains unclear. In a paper by Komaki and colleagues, forced expression of the muscle master transcription factor MyoD in mesenchymal C3H10T1/2 progenitors led to an enhancement of BMP-7 induced osteogenesis [30]. While MyoD has been shown to stimulate the pro-osteogenic transcription factor Osx [31], forced MyoD expression alone is insufficient to promote osteogenic differentiation. We therefore hypothesized that MyoD may potentiate BMP signaling via BMP receptor expression. Myoblasts robustly express BMPR-IA and BMPRII [32], and this may underlie their sensitivity to bone signals [33].

In this study we report a detailed comparison of the response of different mesenchymal progenitors to BMP treatment. Specifically, pre-osteoblasts, BMSCs, skeletal myoblasts, and fibroblasts were examined in a head-to-head fashion, including a combination of cell lines and primary murine cells. This study also aimed to address whether the capacity of progenitors for osteogenic conversion correlates with the expression of BMPRs, and if forced myogenesis could affect BMPR expression and BMP sensitivity in fibroblasts.

## Methods

### Cell culture

C2C12 myoblasts were cultured in DMEM containing 20% FBS while NIH/3T3 fibroblasts were cultured in DMEM containing 10% FBS. MC3T3-E1 pre-osteoblasts were grown in α-MEM containing 10% FBS.

Primary murine cells were obtained from 8–12 week old C57BL6/J mice in a study approved by the CHW animal ethics committee. Primary myoblasts were isolated from mouse hindlimb muscles and enzymatically digested with 0.25% pronase (Merck Sharp & Dohme, Granville, NSW, Australia) for 1 hr at 37°C. Digestion was terminated by the addition of DMEM containing 10% horse serum (Invitrogen, Carlsbad, CA, USA). Isolated cells were sequentially filtered through a 100 μm and 40 μm cell strainers and purified based on a previously published pre-plating technique [34]. Primary myoblasts were cultured in DMEM containing 20% FBS. Primary fibroblasts were obtained by a similar digestion process from fascia adjacent to skeletal muscle and grown in DMEM containing 10% FBS. Primary BMSCs were selected to represent a less-differentiated mesenchymal progenitor population (as opposed to neonatal calvarial osteoblasts, which exhibit a more mature osteoblastic phenotype). BMSCs were isolated from mouse femurs by flushing the bone marrow from the medullary canal with DMEM using a 21 gauge needle. After 3 days of culture in DMEM containing 10% FBS, non-adherent cells were removed by washing and aspiration. Primary cells and cell lines were cultured with antibiotics consisting of 100 unit/ml penicillin and 0.1 mg/ml streptomycin and grown in humidified chambers at 37°C with 5% CO_2_.

### Inducing osteogenic differentiation

Cells were plated in triplicate at a density of 1 × 10^4 ^cells/well in 48-well collagen-coated plates and osteogenic differentiation was induced by culturing cells in osteogenic media (MC3T3-E1 growth media supplemented with 50 μg/ml ascorbic acid and 10 mM β-glycerophosphate). Cells were grown either with 0 or 200 ng/ml BMP-2 (Kamiya Biotech, Thousand Oaks, CA, USA), with media changes every second day.

### Cell viability and alkaline phosphatase (AP) activity assay

Cellular viability was determined using the CellTitre 96 Aqueous One Solution Cell Proliferation Assay kit (Promega, Madison, WI, USA) according to the manufacturer's instructions. AP activity in cells was detected using *p*-nitrophenylphosphate (*p*NPP) (Sigma-Aldrich, St Louis, MO, USA) as the substrate. Cells were fixed with 4% paraformaldehyde (PFA) for 15 min and washed with AP wash buffer (0.1 M NaCO_3 _pH 10, 1 mM MgCl_2_). Cells were then incubated with 10 mM *p*NPP and the reaction left to incubate for 20 min at 37°C. Absorbance was read at 405 nm and the enzyme activity was normalized to cell number obtained from viability assays. All samples were assayed in triplicate. Results are representative of a minimum of two independent experiments.

### Histochemical staining

Mineralization of calcium deposits was assessed by Alizarin Red S staining. Cultures were fixed with 4% PFA for 15 min at room temperature and then stained with Alizarin Red S for 10 min (40 mM, pH 4.2). Non-specific staining was removed by several washes with distilled water.

### RNA extraction, cDNA preparation, and real-time quantitative PCR (qPCR)

Total cellular RNA was extracted from cells grown in 6-well plates using TRIZOL reagent (Invitrogen) as per the manufacturer's instructions. cDNA was then reverse-transcribed from equivalent amounts of total RNA using Superscript III Reverse Transcriptase (Invitrogen). The PCR primer sequences used for amplification are listed in Table 1. All samples were amplified using the SYBR Green PCR reagent kit (Integrated Sciences, Chatswood, NSW, Australia) according to the manufacturer's protocol. PCR was performed on the Rotor-Gene 3000 (Corbett Life Science, Sydney, NSW, Australia).

**Table 1:** Oligonucleotide sequences for quantitative PCR (qPCR)

Target cDNA sequence	Sense primer	Anti-sense primer
*MyoD*	5'-CGGCTACCCAAGGTGGAGAT-3'	5'-ACCTTCGATGTAGCGGATGG-3'
*Bmpr-1a*	5'-ACATCAGATTACTGGGAGCC-3'	5'-TATAGCAAAGCAGCTGGAG-3'
*Bmpr-2*	5'-GTGCCCTGGCTGCTATGG-3'	5'-TGCCGCCTCCATCATGTT-3'
*Gapdh*	5'-GCCACCCAGAAGACTGTGGATGGC-3'	5'-CATGTAGGCCATGAGGTCCACCAC-3'
*Ap*	5'-GGGACTGGTACTCGGATAACGA-3'	5'-CTGATATGCGATGTCCTTGCA-3'
*Ocn*	5'-CGGCCCTGAGTCTGACAAA-3'	5'-GCCGGAGTCTGTTCACTACCTT-3'

All samples were initially denatured at 94°C for 2 min followed by 40 amplification cycles for *Ap*, *Ocn*, and *MyoD *(95°C for 15 s, 60°C for 60 s, and 72°C for 60 s); 55 amplification cycles for *Bmpr-1a*, and *Bmpr-2 *(94°C for 60 s, 55°C for 60 s, and 72°C for 60 s); 35 cycles for *Gapdh *(94°C for 30 s, 55°C for 60 s, and 72°C for 60 s) and extension at 72°C for 60 s. PCR reactions were performed in triplicates and normalized to the housekeeping gene *Gapdh*, to control for cDNA loading. Data are presented as mean fold induction (± SE).

### BMPR promoter analysis

The procedure used to identify potential myogenic regulatory motifs was employed over a region encompassing 2 kb upstream of the BMPR1A transcription start site. This region of interest was identified and extracted in both mouse and human using the UCSC genome browser [35]. A position-specific scoring matrix tool, MatInspector [36], was then used to locate putative binding sites for myogenic transcription factors MyoD, MEF2, MYF3, MYF5 and Myogenin. Low confidence matches were filtered out, retaining only those corresponding to a matrix similarity score of greater than 0.9.

### Forced myogenesis in NIH/3T3 fibroblasts

Lentivirus vectors expressing eGFP (LV-eGFP) or the myogenic transcription factor MyoD (LV-MyoD) under the control of the cytomegalovirus (CMV) promoter were generated using published methods [37]. NIH/3T3 fibroblasts were transduced at a multiplicity of infection (MOI) of 50 in the presence of 8 μg/ml Polybrene (Sigma-Aldrich) overnight. Fresh media was added 18–24 hours following transduction and cells were cultured for a further 2 days before cells were treated with osteogenic media with 200 ng/ml BMP-2 for another 3 days. At the end of the culture period, RNA samples were purified for qPCR analysis.

### Statistical analysis

Assays were conducted at least in triplicate. Values are presented as mean ± standard error (SE), and statistical comparisons were made using a two-tailed student's t-test. P values <0.05 were considered statistically significant.

## Results

### BMP-2 stimulates osteogenesis in myoblastic but not fibroblastic cell lines

The C2C12 and NIH/3T3 cell lines were used to examine the capacity of myoblasts and fibroblasts to undergo BMP-induced osteogenic differentiation respectively. The pre-osteoblastic MC3T3-E1 cell line is not only BMP-responsive but readily undergoes osteogenic differentiation in the absence of BMP treatment, and was used as a positive control. Cells were cultured in osteogenic media and treated cells received 200 ng/ml of BMP-2. *Ap *gene expression, AP staining and AP activity assays were measurements for early bone marker expression, while *Ocn *gene expression and mineralized matrix staining were used as indicators for mature bone differentiation.

**Figure 1:**
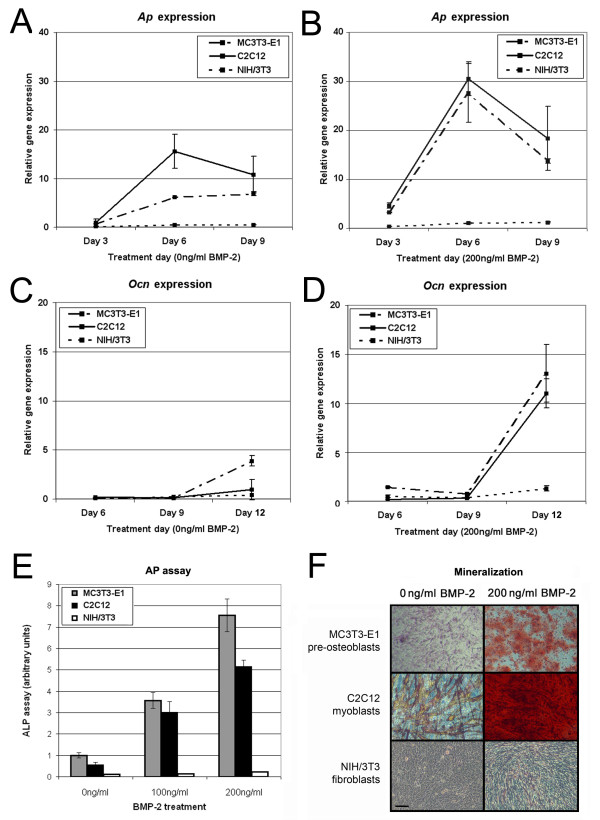
**The effect of BMP-2 on the osteogenic expression in cell lines**. Osteogenic gene expression was examined in MC3T3-E1 (dot-dash line), C2C12 (straight line) and NIH/3T3 (dotted line) cell lines using qPCR with the data normalized to the housekeeping gene *Gapdh*. *Ap *expression was measured as an early osteogenic marker in cells grown in the absence (**A**) or the presence (**B**) of BMP-2. *Ocn *gene expression was measured as a late osteogenic marker in cells cultured in the absence of BMP-2 (**C**) or with BMP-2 added (**D**). In addition, the dose- and cell-dependent effect of BMP-2 on AP activity after 6 days (**E**) and mineralized nodule formation after 12 days (**F**) was measured in all three cell lines. Scale bar = 100 μm.

The addition of BMP-2 increased *Ap *mRNA levels in both MC3T3-E1 pre-osteoblasts and C2C12 myoblasts (Fig 1A–B). By day 9, *Ap *mRNA in BMP-2 treated MC3T3-E1 and C2C12 cells decreased to levels that were similar to those of the untreated cells (Fig 1B). In contrast, NIH/3T3 cells continued to upregulate *Ap *expression up to and after 9 days in culture (Fig 1B). BMP-2 had no significant effect on *Ocn *transcript levels early in culture (Fig 1C–D). In all cell types, *Ocn *expression increased with prolonged culturing. Notably, BMP-2 upregulated C2C12 *Ap *and *Ocn *mRNA to a level comparable to that of BMP-2 treated MC3T3-E1 cells.

In MC3T3-E1 and C2C12 cells, the addition of BMP-2 increased AP activity early on (Fig 1E) and mineralized matrix staining at late time points (Fig 1F). BMP-2 treatment increased osteogenic markers in these cells in a dose-dependent manner. This increase in myoblasts was accompanied by a concomitant downregulation in myogenic expression as seen by immunofluorescent staining for desmin and MyoD (data not shown). In contrast, the NIH/3T3 fibroblasts showed negligible AP activity and did not generate mineralized nodules over the same culture period. Furthermore, NIH/3T3 fibroblasts cells acquired only sporadic AP positive cells after 18 days of BMP-2 treatment and failed to generate a mineralized matrix even after 24 days in culture (data not shown)

### BMP-2 stimulates osteogenesis in primary cells from muscle tissue but not muscle fascia

Myoblastic cells capable of spontaneously differentiating and fusing into myotubes were generated by a standard method involving enzymatic digestion of mouse muscle tissue [34]. This method generated cultures possessing >80% desmin positive myoblasts capable of fusing into multinucleated myotubes. Primary cells were also cultured from muscle fascia. In contrast to the muscle-derived cells, fascial cells developed a stellate morphology and failed to express desmin (data not shown). As controls, BMSCs, which are known to be osteocompetent, were obtained by flushing the femoral marrow cavity and removing non-adherent cells at 3 days after plating.

**Figure 2:**
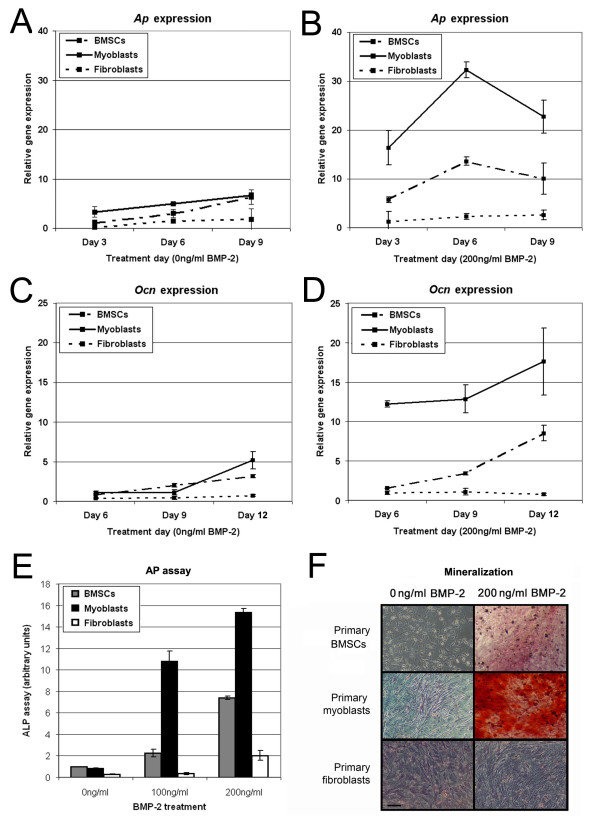
**The effect of BMP-2 on the osteogenic expression in primary-derived cells**. Osteogenic gene expression was examined in primary BMSCs (dot-dashed line), primary myoblasts (straight line) and primary fibroblasts (dotted line) using qPCR and data normalized to the housekeeping gene *Gapdh*. *Ap *expression was measured as an early osteogenic marker in cells cultured in the absence (**A**) or the presence (**B**) of BMP-2. *Ocn *expression was measured as a late osteogenic marker in cells grown in the absence (**C**) or the presence (**D**) of BMP-2. In addition, dose- and cell-dependent effect of BMP-2 on AP activity after 6 days (**E**) and mineralized nodule formation after 12 days (**F**) was measured in all three cell lines. Scale bar = 100 μm.

BMSCs endogenously expressed osteoblast-specific genes, and these were further elevated when cells were cultured with BMP-2 (Fig 2). Untreated primary myoblasts expressed levels of *Ap *and *Ocn *similar to those of untreated BMSCs, which were upregulated with the addition of BMP-2. Elevated levels of *Ap *in primary myoblasts persisted for 6 days and then gradually declined (Fig 2A–B) such that *Ap *expression was below the level of detection by 18 days (data not shown). The decrease in early bone markers was concomitant with an upregulation in the late marker *Ocn *(Fig 2C–D). Patterns of *Ap *and *Ocn *expression were comparable between BMP-treated primary myoblasts and BMSCs. Fascial fibroblasts treated with BMP-2 expressed very low levels of bone-specific mRNAs and no significant increase in *Ap *and *Ocn *transcripts was observed.

BMP-2 induced robust AP expression (Fig 2E) and mineralization staining (Fig 2F) in primary myoblasts that were superior even compared to cultured BMSCs. In contrast, very few AP positive cells were seen in primary fibroblast cultures treated with BMP-2 and no matrix mineralization was observed. The addition of BMP-2 to primary myoblasts resulted in decreases in desmin and MyoD expression, similar to that observed for C2C12 cells (data not shown).

### Bioinformatics analysis of the BMPR promoter regions

**Figure 3:**
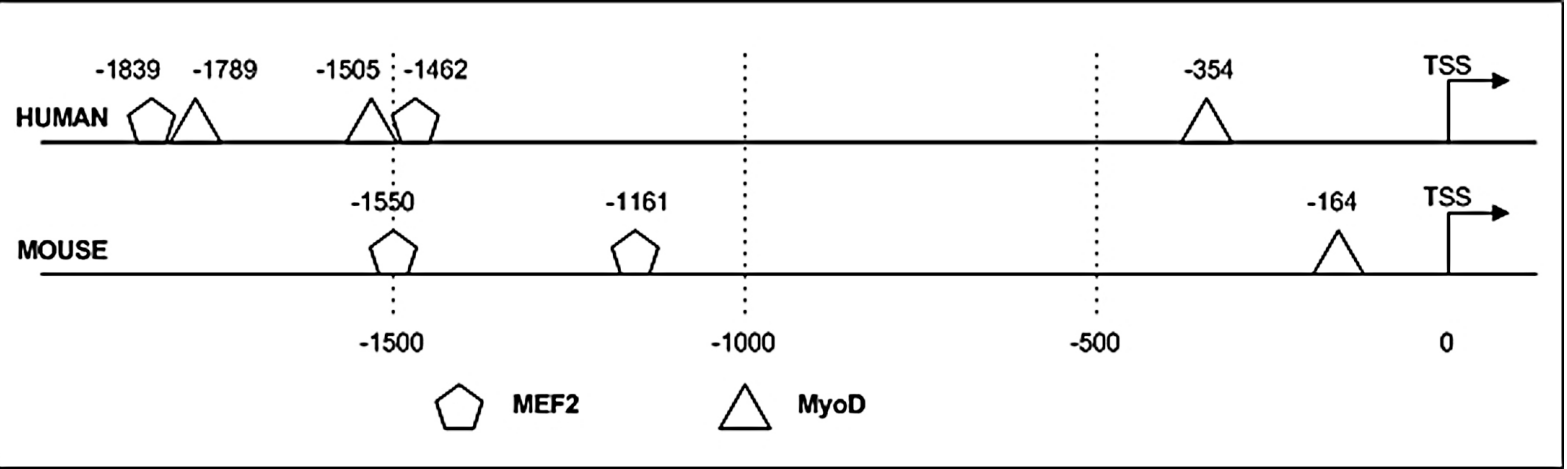
**Analysis of the human and murine BMPR-1A/*Bmpr-1a *promoter region**. Putative myogenic transcription factor binding sites were identified by MatInspector in human and murine *BMPR-1A/Bmpr-1a *promoter regions. Putative MEF2 and MyoD binding sites were predicted within a 2 kb region upstream from the *BMPR-1A *transcriptional start site; putative MEF2 and MyoD were also predicted in the corresponding 2 kb region upstream from the *Bmpr1a *transcriptional start site. The transcriptional start site was inferred from Genbank entries NM_004329 (human) and NM_009758 (mouse). Low confidence and non-myogenic predictions are not shown.

Based on the high relative BMP-responsiveness of myogenic cells, the upstream promoter regions of the BMPRs were examined for myogenic regulatory elements. Analysis was performed using MatInspector, a software package that identifies transcriptional binding sites which uses advanced filtering to generate high-quality matches. A number of high-confidence myogenic factor binding sites were identified in mouse and human *Bmpr-1a/BMPR-1A *promoter regions. In the mouse, this corresponded to one MyoD and two MEF2 sites, and in the human this corresponded to three MyoD and two MEF2 sites (Fig 3). The expected number of occurrences due to chance alone over a 2 kb region was 0.26 and 0.06 for the MyoD and MEF2 motifs in both mouse and human, respectively. Thus the observed number of occurrence(s) for each motif was always greater than the expected species-specific occurrence value, denoting that sufficiently stringent criteria were employed to identify putative transcription factor binding sites. Several Smad-binding consensus sequences were also detected (data not shown), indicating the potential involvement of alternative non-myogenic regulatory elements.

Analysis of the murine *Bmpr-1b *and *Bmpr-2 *2 kb promoter sequence identified a single MyoD site in each. Based on the expected number of false positives, identification of these sites was not sufficient to establish myogenic responsiveness.

### Osteogenic sensitivity correlates with *Bmpr-1a *expression

**Figure 4:**
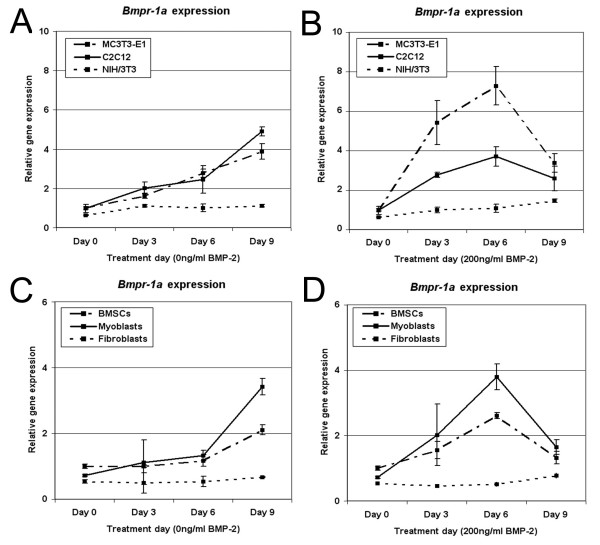
**Cellular response to BMP-2 correlates to theexpression levels of *Bmpr-1a***. *Bmpr-1a *expression levels were examined using qPCR and normalized to the housekeeping gene *Gapdh*. Values were generated for cell lines grown in the absence of BMP-2 (**A**) or with BMP-2 added (**B**), and in mouse primary derived cells grown without (**C**) or with (**D**) BMP-2 treatment. *Bmpr-1a *expression increased over time when cells were grown in osteogenic media, and were even greater under BMP-2 stimulation. The lowest *Bmpr-1a *levels were observed in NIH/3T3 cells and primary fibroblasts, which were previously shown to be the least sensitive to BMP-2 treatment.

To further explore BMPR expression as a mechanism for modulating osteogenic differentiation, gene expression was examined in cell lines and primary cells. *Bmpr-1a *expression was readily detectable by qPCR. Basal levels of the *Bmpr-1a *gene in NIH/3T3 fibroblasts were found to be significantly lower than in MC3T3-E1 pre-osteoblasts (38% reduction, p = 0.02). No significant difference was seen between C2C12 myoblasts and MC3T3-E1 cells. *Bmpr-1a *expression was further upregulated in these lines cultured in osteogenic media, either in the absence or presence of BMP-2 (Fig 4A–B). Notably, C2C12 cells treated with BMP-2 eventually down-regulated their *Bmpr-1a *expression by day 9; this was also evident in MC3T3-E1 cells. In contrast, NIH/3T3 cells did not significantly upregulate their *Bmpr-1a *expression with BMP-2 treatment. In NIH/3T3 fibroblasts, *Bmpr-1a *was only substantively elevated after 21 days in culture (5.3-fold higher p = 0.002 compared to day 0 cells) (data not shown). In primary cell cultures, comparable results were observed (Fig 4C–D).

### Forced expression of MyoD in fibroblasts enhances *Bmpr-1a *expression

**Figure 5:**
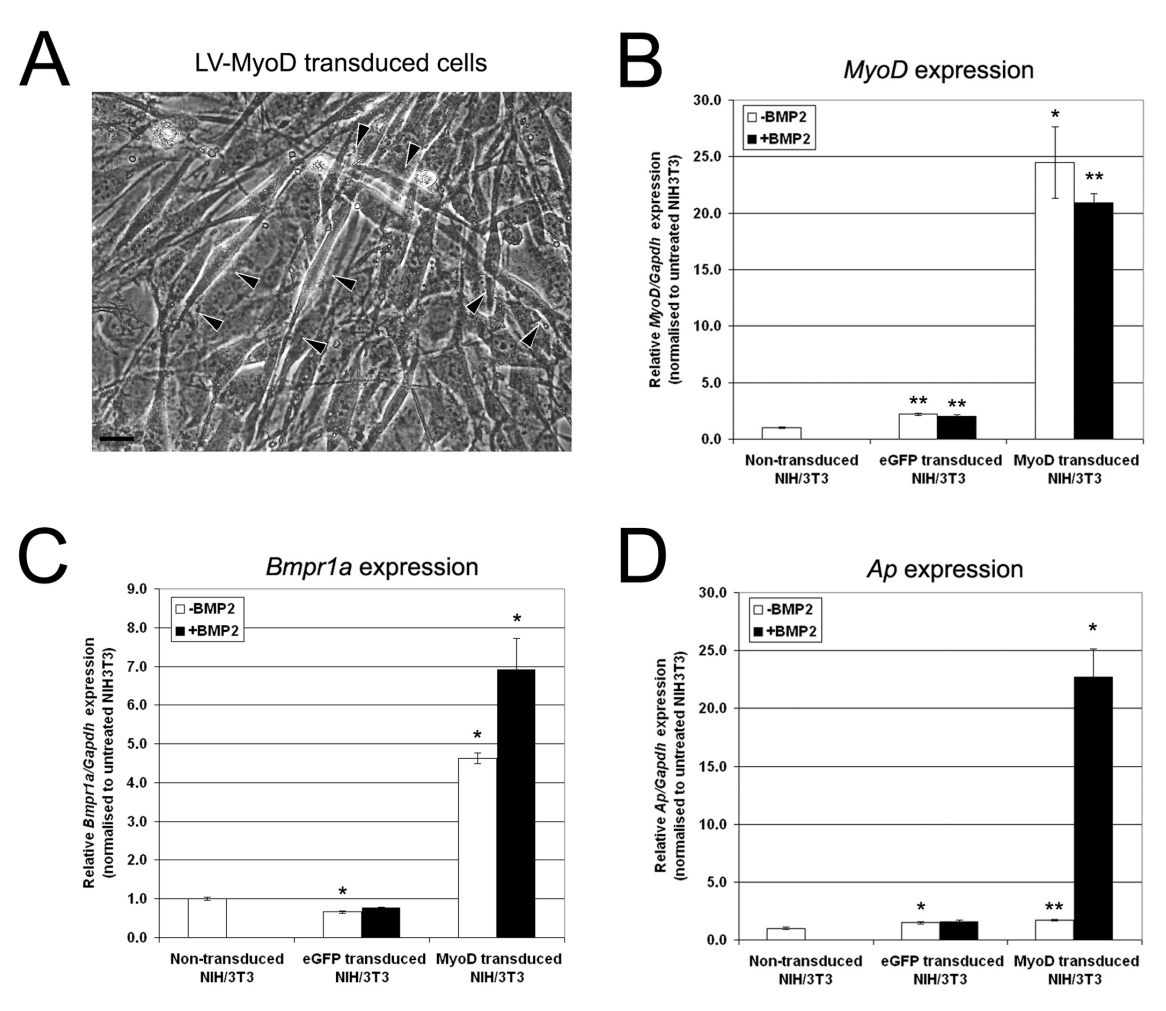
**Forced MyoD expression in NIH/3T3 fibroblasts increases *Bmpr-1a *expression**. NIH/3T3 cells were transduced using LV-MyoD and LV-eGFP lentiviral vectors. Fibroblasts transduced with LV-MyoD developed an elongated morphology that resembled myotubes (**A**). Scale bar = 50 μm. Three days after the initial transduction, cells were cultured for a further 3 days in the presence or the absence of BMP-2. Gene expression for *MyoD *(**B**), *Bmpr-1a *(**C**) and *Ap *(**D**) were measured using qPCR, relative to *Gapdh*. Comparative expression analysis was performed relative to non-transduced, untreated NIH/3T3 fibroblasts. The majority of values were statistically significant (*p < 0.05, **p < 0.01), however, substantive changes in gene expression (>4-fold increase) were only observed in LV-MyoD transduced cells.

Prior experiments indicated that *Bmpr-1a *was robustly expressed by myoblasts but not fibroblasts. To determine whether myogenic factors were important for modulating Bmpr-1a expression, we forced fibroblasts to differentiate down the myogenic lineage using a MyoD-expressing lentiviral vector (LV-MyoD). To account for any adverse effects on cell signaling mediated by lentiviral transduction, an equivalent eGFP lentiviral vector (LV-eGFP) was included as a control. Viral transduction was efficient and high levels of eGFP were detected in a majority (>70%) of the cells 72 hrs post-transduction (data not shown). Critically, NIH/3T3 fibroblasts transduced with the LV-MyoD construct fused and formed myotubes demonstrating successful induction of a myogenic differentiation program (Fig 5A). A 20–25 fold increase in MyoD expression was also confirmed by qPCR (Fig 5B).

Three days after transduction, eGFP- and MyoD-expressing NIH/3T3 cells were cultured for an additional 3 days in growth media or osteogenic differentiation media supplemented with BMP-2. Analysis by qPCR revealed a 4.6 fold increase in *Bmpr-1a *expression in MyoD-expressing NIH/3T3 cells without BMP-2 (p = 0.02) and a 6.9 fold increase in MyoD-expressing NIH/3T3 cells treated with BMP-2 (p = 0.02) (Fig 5C). *Ap *expression was then measured as an indicator of osteogenesis. *Ap *expression in MyoD-transduced fibroblasts grown in growth media was comparable to levels seen in non-transduced cells and LV-eGFP transduced controls. However, when LV-MyoD transduced fibroblasts were treated with BMP-2, *Ap *expression was elevated 23 fold (p = 0.01) compared to non-transduced controls (Fig 5D). Thus, forced myogenesis and exogenous BMP-2 treatment acted synergistically in fibroblasts to upregulate *Ap *expression.

## Discussion

This study represents a controlled head-to-head comparison of the responsiveness of different progenitor lineages to BMP-2. While prior studies have illustrated that BMP-2 can induce an osteogenic phenotype in myogenic cells, we have addressed the relative BMP-2 sensitivity of myoblasts relative to fibroblasts and osteoprogenitors. We hypothesized that myoblasts would show a strong osteogenic response, particularly when compared to fibroblasts. Considering the pathological complications associated with fibrosis in orthopaedic repair, the sensitivity of fibroblasts to osteogenic stimulation is highly relevant.

Our data clearly confirms the body of literature showing that BMP-2 treatment readily induces osteogenic differentiation in myoblasts [15,16]. Consistent with prior studies, we observed an associated reduction in myogenic markers in myoblasts undergoing osteogenesis [29]. Our findings support our initial hypothesis that the osteogenic potential of myoblasts when exposed to osteogenic signaling is comparable to that of standard osteoprogenitors and far exceeds that of fibroblasts. Finally, we speculated that the sensitivity of cells to BMP-2 may reflect the expression of BMPRs on the cell surface. Previous reports have shown that BMPR-IA but not BMPR-1B is robustly expressed in C2C12 myoblasts [32]. Transfection of a dominant-negative BMPR-1A into BMP-2 treated C2C12 cells inhibited AP expression. This was partially rescued by the transfection of wild-type BMPR-1A into C2C12 cells over-expressing the truncated BMPR-1A mutant. In contrast, truncated BMPR-IB had no effect on BMP-2 signaling in C2C12 cells. More recently, a siRNA knockdown of BMPR-1A has been shown to inhibit BMP-2 mediated osteogenic differentiation in human mesenchymal stem cells [33].

We observed strong *Bmpr-1a *expression in myoblasts and osteoprogenitors that could be enhanced by BMP-2 treatment, while fibroblasts failed to express robust levels of *Bmpr-1a*. In addition, fibroblasts transduced with the myogenic transcription factor MyoD upregulated their *Bmpr-1a *expression. Although these data represent a strong correlation between *Bmpr-1a *expression and BMP sensitivity, the mechanism underlying the varying osteogenic potential of different cell lineages may be more complex. Forced osteogenesis experiments using Runx2 expression constructs revealed that fibroblasts have a reduced capacity for mineralization that is likely to be independent of extracellular BMP signaling [38].

While we have observed a consistent upregulation of osteogenic markers, our data does not fully resolve whether genuine transdifferentiation has taken place. While myogenic cells treated with BMP-2 progressed from expressing early differentiation markers (*Ap*/AP expression) to late markers (*Ocn *expression, matrix mineralization), osteogenic stimulation was maintained during this time. When Katagiri *et al*. treated C2C12 cells for 6 days with BMP-2 stimulation and then reverted to a myogenic growth media, cells were found to revert to a myogenic profile [15]. This would suggest that *bone fide *transdifferentiation did not occur, and that muscle progenitors retained a "memory" of their original myogenic lineage. However, it is unclear if cells are treated with BMP for longer, whether a myogenic memory would remain. Moreover, it is possible that returning cells to a myogenic media preferentially selected for the adherence, survival, and proliferation of cells that had not yet committed to an osteogenic status. Finally, the myogenic reversion of BMP-2 treated cells *in vitro *poorly models the *in vivo *situation where a muscle progenitor may become exposed to persistent osteogenic signals in a bone environment.

Although our results suggest that muscle progenitors have a high responsiveness to BMP signaling, it does not rule out the possibility that alternative cell populations may contribute to bone formation in muscle. This includes circulating osteoprogenitors originating from the bone compartment, as irradiation of the bone marrow compartment can attenuate ectopic bone formation [39]. However, irradiation of progenitors in the soft tissues was found to be equally effective, suggesting that local progenitor cells are also of importance [40]. Vascular mesoangioblasts and pericytes have been a focus of recent interest due to their potential for muscle cell therapy [41]. While mesoangioblasts are of an endothelial lineage, pericytes have been shown to not express vascular markers [42,43]. Although pericytes have been recently shown to be a sub-population of progenitors with significant myogenic potential, they do not express MyoD when freshly isolated [42]. Thus between multi-lineage progenitor cells such as mesoangioblasts, side-population cells and muscle-derived stem cells, pericytes, and classical satellite cells, the milieu of progenitor cells found in muscle tissue is complex and ever expanding. The relative capacity of these different subpopulations of myogenic progenitors to respond to osteogenic stimulation has yet to be explored, however we hypothesize that assaying for BMPR expression will be an effective predictive measure. Based on the presence of multiple myogenic-responsive elements in the *Bmpr-1a/BMPR-1A *promoter sequences, we anticipate that all of these cell types will robustly express *Bmpr-1a*.

There remains a possibility that other related receptors may also be involved. In the genetic disease FOP, the ectopic ossification of soft tissues in patients is the result of a dominant mutation in ACVR1/ALK2, a type 1 BMP receptor [44]. Based on molecular modeling, it was hypothesized that this mutation led to destabilization of a region used for binding negative regulators of R-Smad signaling; thus the mutant receptor may be promiscuously activated. Nevertheless, there are no other indications that ACVR1/ALK2 has a normal physiological role in osteogenesis outside of the pathology of FOP. It has also been recently demonstrated that BMP-6 and BMP-7 primarily signal via the ACVR1/ALK2 receptor, while BMP-2 and BMP-4 favor the BMPR-1A receptor [45].

## Conclusion

In conclusion, this study characterizes the osteogenic differentiation profile of committed osteoprogenitors, myogenic cells, and non-myogenic mesenchymal cells both with and without BMP-2 treatment. Consistent with previous reports, myogenic cells readily establish an osteoblastic phenotype and we found this to be concomitant with an upregulation of BMPR expression. Future work will be required to explore whether endogenous myogenic cells are capable of making an *in vivo *contribution to bone formation in situations of ectopic bone formation and bone repair.

We also observed distinct patterns of *Bmpr-1a *expression in response to osteogenic stimulation, which may have physiological significance. *Bmpr-1a *levels also strongly correlated with responsiveness to BMP-2, and thus *Bmpr-1a *expression may be an alternative indicator for a cell's osteogenic potential. This could have practical applications for the bone tissue engineering field where researchers are looking to isolate highly osteogenic cell populations.

## Competing interests

The authors declare that they have no competing interests.

## Authors' contributions

AS, RL, and DGL participated in the design of the study, interpretation of the data and drafted the manuscript. The experimental data was chiefly generated by RL. SGL provided the LV-eGFP and LV-MyoD viral constructs and assisted with virus production. ML performed the bioinformatics analysis. All authors read and approved the final manuscript.

## Pre-publication history

The pre-publication history for this paper can be accessed here:


